# Acidic Electrolyzed Water as a Novel Transmitting Medium for High Hydrostatic Pressure Reduction of Bacterial Loads on Shelled Fresh Shrimp

**DOI:** 10.3389/fmicb.2016.00305

**Published:** 2016-03-09

**Authors:** Suping Du, Zhaohuan Zhang, Lili Xiao, Yang Lou, Yingjie Pan, Yong Zhao

**Affiliations:** ^1^College of Food Science and Technology, Shanghai Ocean UniversityShanghai, China; ^2^Shanghai Engineering Research Center of Aquatic-Product Processing and PreservationShanghai, China; ^3^Laboratory of Quality and Safety Risk Assessment for Aquatic Product on Storage and Preservation (Shanghai), Ministry of Agriculture ShanghaiShanghai, China

**Keywords:** acidic electrolyzed water, high hydrostatic pressure processing, indigenous microflora, inactivation, aquatic products

## Abstract

Acidic electrolyzed water (AEW), a novel non-thermal sterilization technology, is widely used in the food industry. In this study, we firstly investigated the effect of AEW as a new pressure transmitting medium for high hydrostatic pressure (AEW-HHP) processing on microorganisms inactivation on shelled fresh shrimp. The optimal conditions of AEW-HHP for *Vibrio parahaemolyticus* inactivation on sterile shelled fresh shrimp were obtained using response surface methodology: NaCl concentration to electrolysis 1.5 g/L, treatment pressure 400 MPa, treatment time 10 min. Under the optimal conditions mentioned above, AEW dramatically enhanced the efficiency of HHP for inactivating *V. parahaemolyticus* and *Listeria monocytogenes* on artificially contaminated shelled fresh shrimp, and the log reductions were up to 6.08 and 5.71 log_10_ CFU/g respectively, while the common HHP could only inactivate the two pathogens up to 4.74 and 4.31 log_10_ CFU/g respectively. Meanwhile, scanning electron microscopy (SEM) showed the same phenomenon. For the naturally contaminated shelled fresh shrimp, AEW-HHP could also significantly reduce the micro flora when examined using plate count and PCR-DGGE. There were also no significant changes, histologically, in the muscle tissues of shrimps undergoing the AEW-HHP treatment. In summary, using AEW as a new transmitting medium for HHP processing is an innovative non thermal technology for improving the food safety of shrimp and other aquatic products.

## Introduction

Shrimp is one of the most popular and important seafood worldwide because of its delicacy and abundant nutrients (Norhana et al., 2010). In China, consumption of shrimp, especially shelled fresh shrimp, has increased annually (Wang et al., 2013). However, shrimp products are usually contaminated with numerous spoilage organisms and pathogens (McCarthy, 1997; Liu et al., 2006; Xie et al., 2012). These bacteria were regarded as the crucial influencing factors for the quality and safety of shrimp, and could easily increase the possibilities of spoilage and infection risk (Nirmal and Benjakul, 2010). Therefore, development of an efficient and international accepted intervention method for eliminating these microorganisms in shrimp products would be of great value to the seafood industry and for improving public health (Lenz et al., 2015; Sevenich et al., 2015).

A variety of intervention methods have been established for controlling spoilage or pathogenic bacteria in food, such as high hydrostatic pressure, electrolyzed oxidizing water, ultrasound, irradiation, some chemicals or essential oils (McCarthy, 1997; Andrews et al., 2000; Mahmoud, 2009; Chen et al., 2011; Ma and Su, 2011; Prakash et al., 2012). High hydrostatic pressure (HHP), a non-thermal food sterilization and preservation technology, could reduce microorganisms without damaging the sensory quality of the products (Rendueles et al., 2011; Belletti et al., 2013a; Lorido et al., 2015). In a typical HHP procedure, the food products are sealed and placed into a steel chamber containing a pressure transmitting medium, and pumps are used to create pressure. The effect of HHP for food sterilization and preservation could be influenced by the treatment pressure, treatment time and pressure transmitting medium (Georget et al., 2015). Traditionally, common water is often used as the pressure transmitting medium for HHP (Rubio et al., 2013; Omer et al., 2015; Ma and Mu, 2016), however some researches also suggested that change of pressure transmitting fluids might improve the inactivation effect of HHP (Balasubramanian and Balasubramaniam, 2003; Georget et al., 2015).

In recent years, acidic electrolyzed water (AEW) has been regarded as an effective sanitizer and widely used in the food industries of many countries (Smigic et al., 2009; Wang et al., 2014). By electrolysis in the specific device, a diluted sodium chloride solution dissociates into AEW, which has a low pH of 2–3, a high oxidation-reduction potential (ORP) of > 1100 mV, and an active chlorine content (ACC) of 10–90 ppm (Hricova et al., 2008). In contrast with conventional sanitizer, AEW not only has better bactericidal effect, but also less adverse impact on public health and the environment (Katayose et al., 2007; Huang et al., 2008; Xie et al., 2012). Previous research has proven that AEW in combination with other intervention methods could significantly enhance their inactivation efficiency (Liu R. et al., 2013). However, no studies have reported that AEW could be used as a new transmitting medium in HHP. Also, the impact of AEW on the inactivation efficiency of HHP is unknown.

This study firstly developed a novel sterilization and preservation technology by using the HHP combined with AEW as a new transmitting medium (AEW-HHP). A response surface (RS) model was applied to quantify the influence of AEW on high hydrostatic pressure processing for reducing pathogenic bacteria on shelled fresh shrimp. And the preservation effect of this new technology was evaluated by the reduction of indigenous microflora and the change of muscle fiber of shelled fresh shrimp. This novel sterilization and preservation technology could provide a powerful tool for eliminating pathogenic microorganisms and improving the seafood quality and safety.

## Materials and methods

### Bacterial strain and culture preparation

A four-strain cocktail of *V. parahaemolyticus* strains (ATCC 17802; ATCC 33847; F 18, *Litopenaeus vannamei* isolate; F 36, serotype O3: K6) and a two-strain cocktail of *L. monocytogenes* (ATCC 19115; ATCC 19116) were used in the study. Each strain for *V. parahaemolyticus* (stored in 25% glycerol at -80°C) was separately cultured in tryptic soy broth (TSB, Beijing Land Bridge Technology Company Ltd., Beijing, China) plus 3% NaCl and incubated at 37°C for 18 h. Other strains of *L. monocytogenes* were grown in TSB at 37°C for 18–20 h. Enriched cultures were pooled into a sterile centrifuge tube and centrifuged at 3000 g, 25°C for 10 min (Centrifuge 5417R, eppendorf, Germany). Pelleted cells were re-suspended in sterile peptone water (PW; 0.85% NaCl, 0.1% peptone) to obtain a multi-strain cocktail of ~ 9 log_10_ CFU/mL of *V. parahaemolyticus* and *L. monocytogenes*, respectively.

### Preparation and inoculation of shrimp samples

Fresh shrimps (*Litopenaeus vannamei*) were purchased alive from a local market in Shanghai, PR China before each experiment and transported to the laboratory in plastic bags containing pond water sparged with oxygen to keep the shrimps viable. At the laboratory the shrimps were rinsed with running tap water, a protocol described in GB/T 4789.7-2008 (Code of National Standard of China, 2008). The shrimps were beheaded, peeled, and sterilized under UV light for 30 min. Shelled fresh shrimp samples (7 ± 1 g per shrimp) were soaked into 200 mL of suspensions containing four *V. parahaemolyticus* strains (or two *L. monocytogenes* strains) for 30 min, and then transferred to plastic plates for 30 min to allow for bacterial attachment. The above protocol was carried out at room temperature (23 ± 2°C). The inoculated shrimps were used for the subsequent treatments.

### Preparation of acidic electrolyzed water (AEW)

AEW with different physicochemical characteristics was prepared by electrolyzing different concentrations of sodium chloride (NaCl concentration to electrolysis; Table 1) using an AEW generator (FW-200, AMANO, Japan). The generator was allowed to run for 15 min with the amperage setting as 10 A before collecting water. The pH and ORP were determined using a pH/ORP meter (model pH 430, Corning Inc., NY). The ACC in AEW was determined by a colorimetric method using a digital chlorine test kit (RC-2Z, Kasahara Chemical Instruments Corp., Saitama, Japan). All measurements were carried out in triplicate. The AEW had a range of pH 2.28–2.39, ORP 1077–1168 mV, and ACC 36–73 mg/L.

**Table 1:** **Code and level of variables used for the Box-Behnken experimental design**.

**Levels**	**Variables**		
	***X*_1_ NaCl concentration (g/L)**	***X*_2_ Pressure (MPa)**	***X*_3_ Time (min)**
−1	1.0	200	5
0	1.5	300	10
1	2.0	400	15

### High hydrostatic pressure (HHP) treatment

A high pressure food processor (HPP.L2-600/2, Tianjin Tyson Miaotuo Biological Engineering Technology Company Ltd., Tianjin, China) was used in the present study. The come-up rate was approximately 240 MPa/min and the pressure release time was almost immediate. Samples were packaged in sterile plastic bags and treated under the desired levels 200, 300, or 400 MPa, for treatment time 5, 10, or 15 min. Common water or AEW was used as the transmitting medium for HHP.

### Bacterial enumeration

The bacterial enumeration method used in this study was the plating counting method according to previous studies (Belletti et al., 2013a; Wang et al., 2014). Briefly, shrimp samples were homogenized for 2 min in a stomacher (BagMixer400VW, Interscience, France). Then, a direct-plating procedure was used for the enumeration of *V. parahaemolyticus, L. monocytogenes* and indigenous microflora on shrimp samples. TCBS ager for *V. parahaemolyticus*, PALCAM agar (Beijing Land Bridge Technology Company Ltd., Beijing, China) for *L. monocytogenes* and Trypticase Soy Agar (TSA; Beijing Land Bridge Technology Company Ltd., Beijing, China) for indigenous microflora was used, respectively. The detection limit of the direct-plating was approximately 2 log_10_ CFU/g. Colonies were counted after the plates were incubated at 37°C for 24 h. Three replicates at each sampling time were done.

### Response surface model development and validation

RS methodology based on the Box-Behnken experimental design (BBD) was used to describe the relationship and interactions between three technological variables (Table 1) on the inactivation of *V. parahaemolyticus*. Design Expert package (Version 8.0.6, Stat-Ease Inc., Minneapolis, USA) was used to design the treatment conditions (Table 2), for regression analysis and to generate the second order polynomial model. The shrimp samples were treated under different conditions (Table 2), and the response value was expressed as the Log reductions after the treatments.

**Table 2:** **Actual and predicted reduction of *V. parahaemolyticus* on shrimps by response surface model according to the Box-Behnken experimental design arrangement**.

**Trial**	**NaCl concentration (g/L)**	**Pressure (MPa)**	**Time (min)**	**Bacterial reduction (log_10_ CFU/g)**	
				**Actual value**	**Predicted value**
1	1.0	200	10	2.72 ± 0.067^a^	2.79
2	2.0	200	10	3.32 ± 0.049^b^	3.22
3	1.0	400	10	5.62 ± 0.046^h^	5.73
4	2.0	400	10	5.91 ± 0.066^i^	5.83
5	1.0	300	5	3.73 ± 0.283^c^	3.69
6	2.0	300	5	3.88 ± 0.064^d^	4.02
7	1.0	300	15	4.37 ± 0.042^e^	4.23
8	2.0	300	15	4.41 ± 0.339^e, f^	4.45
9	1.5	200	5	2.83 ± 0.071^a^	2.80
10	1.5	400	5	5.67 ± 0.012^h^	5.60
11	1.5	200	15	3.24 ± 0.099^b^	3.31
12	1.5	400	15	6.03 ± 0.004^i^	6.06
13	1.5	300	10	4.52 ± 0.031^f^	4.57
14	1.5	300	10	4.56 ± 0.011^f, g^	4.57
15	1.5	300	10	4.52 ± 0.048^f^	4.57
16	1.5	300	10	4.56 ± 0.009^f, g^	4.57
17	1.5	300	10	4.68 ± 0.049^g^	4.57

Mean (standard deviation); values with different superscript letters indicate statistical significant differences according to the LSD test.

Analysis of the Variance (*ANOVA*) and the corresponding post-hoc contrasts were performed to compare the results of the different experimental conditions. The statistical significance and goodness of fit of the model were evaluated using the determination coefficients (*R*^2^), the *P*-values from the Fisher *F*-test and the Lack of Fit test. The accuracy of the RS model describing the inactivation of *V. parahaemolyticus* was evaluated by the following criteria: the accuracy factor (*Af*, Equation 1), bias factor (*Bf*, Equation 2) and the root-mean-squares error (RMSE, Equation 3; Zurera-Cosano et al., 2006; Dong et al., 2007):

(1) $$\begin{array}{rcl} Af & = & 10^{\left(\frac{\sum |Log\left(pred∕obs\right)|}{n}\right)} \end{array}$$

(2) $$\begin{array}{rcl} Bf & = & 10^{\left(\frac{\sum Log\left(pred∕obs\right)}{n}\right)} \end{array}$$

(3) $$\begin{array}{rcl} \text{RSME} & = & \sqrt{\frac{\sum {\left(obs-pred\right)}^{2}}{n}} \end{array}$$

where *obs* was observed values, *pred* was predicted values by RS model, and the *n* stands for the number of observations. Ideally, predictive models would have *Af* = *Bf* = 1. The response surface graphs and contour plots were used to describe the individual effects and interactions of the variables.

To validate the model, eight additional trials with random combinations of four variables were conducted (Table 3). The method of bacterial inactivation on shrimps was the same as explained previously for the inactivation treatments. The selected parameters were within the original range of the experimental design but not included in the establishment of the model. The observed results were used to evaluate performance of the model by the *Af* (Equation 1) and the *Bf* (Equation 2) as proposed by Ross (1996).

**Table 3:** **Actual and predicted reduction values for inactivation of *V. parahaemolyticus* on shelled fresh shrimp under the additional random eight conditions**.

**Trial**	**NaCl concentration (g/L)**	**Pressure (MPa)**	**Time (min)**	**Bacterial reduction (log_10_ CFU/g)**	
				**Actual value**	**Predicted value**
1	1.2	350	7	4.51 ± 0.18	4.71
2	1.7	250	7	3.97 ± 0.22	4.04
3	1.2	250	7	3.72 ± 0.31	3.56
4	1.7	350	7	5.25 ± 0.13	5.19
5	1.2	350	12	5.04 ± 0.08	5.11
6	1.7	250	12	4.04 ± 0.31	4.44
7	1.2	250	12	4.12 ± 0.42	3.96
8	1.7	350	12	5.67 ± 0.05	5.67

### Application of AEW-HHP treatment on artificially contaminated shelled fresh shrimp

The optimal conditions of AEW-HHP for inactivation of bacteria on artificially contaminated shrimp were obtained by RS methodology. Based on the optimal conditions, artificially contaminated shelled fresh shrimp samples with *V. parahaemolyticus* or *L. monocytogenes* were treated by AEW-HHP. The treatment conditions were set as NaCl concentration to electrolysis 1.5 g/L, treatment time 10 min and treatment pressure 200, 300, and 400 MPa. Bacterial reduction was determined by plating as described above.

### Observation by scanning electron microscopy (SEM)

After AEW-HHP treatment, bacteria on artificially contaminated shelled fresh shrimp were observed by SEM. The preparation and observation of samples were according to the conventional method (Shen and Zhiguo, 2008). In brief, the treated artificially contaminated shrimp samples were fixed with 2.5% glutaraldehyde, then dehydrated in graded ethanol series, coated with gold-palladium (Ion Sputter and Carbon Coating Unit E-1045, Hitachi Company, Japan) and observed under a scanning electron microscope (NOVA NanoSEM230, FEI Company, USA).

### Application of AEW-HHP treatment on naturally contaminated shelled fresh shrimp

To further verify the inactivation efficacy, the AEW-HHP treatment was applied to eliminate the indigenous microflora on naturally shelled fresh shrimp. The treatment conditions were set as NaCl concentration to electrolysis 1.5 g/L, treatment time 10 min and treatment pressure 200, 300, and 400 MPa. The bacterial enumeration of indigenous microflora was carried out using TSA. The diversity of bacteria in shrimp was analyzed by PCR-DGGE, and the quality of shrimp was evaluated by histological analysis.

### Bacterial diversity analysis using PCR-DGGE

PCR-DGGE analysis was used to assess the changes in the diversity of bacteria on naturally contaminated shrimps. DNA was extracted according to the method described by Ampe et al. (1999). Briefly, all samples were homogenized for 2 min in the stomacher. The resulting suspension (1 mL) was centrifuged (12,000 g, 2 min), and then the supernatant was discarded. The obtained pellet was used to extract DNA using DNA extraction kit (TIANamp bacteria DNA kit, Tiangen Biotech CoLtd., Beijing, PR China). One microliter of the DNA sample was used as template for the PCR. The primers V3-2 (5′-ATTACCGCGGCTGCTGG-3′) and V3-3 incorporated a 40-bp GC clamp (5′-CGCCCGCCGCGCGCGGCGGGCG GGGCGGGGGCACGGGGGGCCTACGGGAGGCAGCAG-3′) which was used to amplify the hypervariable V3 region of the 16S rDNA (Muyzer et al., 1993). The V3 region is a good choice in terms of length and species-species heterogeneity (Wang et al., 2015). The PCR mix was composed of 25 μL of Premix Ex Taq (Takara, Otsu, Japan), 19 μL of ddH_2_O, 1 μL of each primer, and 2 μL of DNA template. The reactions were performed on a thermal cycler (Mastercycler pro S; Eppendorf AG, Germany). The PCR program was performed according to Muyzer et al. (1993) with slight modifications as follows: initial denaturation at 95°C for 3 min; 25 cycles of denaturation at 95°C for 1 min, annealing at 55°C for 1 min and extension at 72°C for 30 s; final extension at 72°C for 5 min. The amplified products were separated with 1% (m/v) agarose gel electrophoresis and visualized under UV light.

DGGE was performed with BioRad DCode™ Universal Mutation Detection System (BioRad Laboratories, USA) using 20 μL of the purified and concentrated PCR product. The PCR products were applied to 8% (m/v) polyacrylamide gels in 1 × TAE buffer which contained a 40–55% urea-formamide denaturing gradient. Electrophoresis was conducted at 100 V for 10 h under a constant temperature (60°C). The DGGE gels were stained with SYBR green I and visualized under UV light (Lin et al., 2013). All determinations were carried out in duplicate.

Scanned images of the DGGE gels were analyzed with Image Lab (Bio-Rad, USA). Shannon index was calculated by DGGE banding pattern analysis. Shannon index of bacterial diversity, *H*′, was obtained by Equation (4). *P*_*i*_ was calculated as Equation (5).

(4) $$\begin{array}{rcl} H^{\prime } & = & -P_{i}\;log\;P_{i} \end{array}$$

(5) $$\begin{array}{rcl} P_{i} & = & n_{i}∕N \end{array}$$

where *P*_*i*_ is the importance probability of the bands in a gel lane, *n*_*i*_ is the height of a peak and *N* is the sum of all the peak heights of the bands in the densitometric profile (Ogino et al., 2001).

### Histological analysis

The histological analysis was performed according to Liu D. et al. (2013) with some modifications. The second uromere of shrimp samples was cut, and fixed in 4% formalin for 24 h. The tissue was dehydrated with a graded series of ethanol (70, 80, 90, 95, and 100%), and then transferred to xylene and embedded in paraffin for 1–2 h. The tissue sections of 7 μm were obtained using a RM2235 microtome (Leica Microsystems CMS GmbH, Wetzlar, Germany). The sections were transferred onto glass slides after reshaped in water, dried, deparaffinized, rehydrated, stained (hematoxylin-eosin staining), and then photo-graphed. All determinations were carried out in triplicate.

An image analysis of each picture was made according to the methods of Díaz-Tenorio et al. (2007). Blank area represents the area of gaps between muscle fibers. The percentage of blank areas in each picture was calculated. Results were expressed as percentages of total analyzed area.

### Statistical analysis

All values were expresses as the mean ± standard deviation (*SD*). Statistical analysis was performed by using SPSS statistical package 17.0 (SPSS Inc., Chicago, USA). One way analysis of variance was conducted to compare the effects under different conditions. The least significant difference (*LSD*) test was used to determine differences at α = 0.05.

## Results

### Optimal conditions of AEW-HHP for *V. parahaemolyticus* inactivation

The initial load on shelled fresh shrimp was approximately 7.1 log_10_ CFU/g. Seventeen trials under different combined conditions (NaCl concentration to electrolysis, treatment pressure and treatment time) were designed by BBD. Table 2 shows the results of Log reductions of *V. parahaemolyticus* on shelled fresh shrimp under different conditions. The actual values of Log reductions ranged from 2.72 to 6.03 log_10_ CFU/g.

A backward stepwise regression was carried out using Design Expert package to develop a RS model based on Table 2, and the established model was as follows:

(6) $$\begin{array}{l} R=-3.8825+3.9955X_{1}+0.011527X_{2}+0.2397X_{3}\\ \;-0.0016X_{1}X_{2}-0.011X_{1}X_{3}-0.000025X_{2}X_{3}-1.046X_{1}^{2}\\ \;+0.00000835X_{2}^{2}-0.00836X_{3}^{2} \end{array}$$

where *R* was response value (log_10_ CFU/g); *X*_1_, *X*_2_, and *X*_3_ represent variables, including NaCl concentration to electrolysis, treatment pressure and treatment time, respectively. The results of the *ANOVA* for quadratic model indicated the goodness of fit of the regression equation: the *R*^2^ and *Adj. R*^2^ were 0.994 and 0.986, respectively. The value of the *Adj.R*^2^ indicated a high degree of correlation between the observed and predicted values, which suggested that only 1.6% of the total variation cannot be explained by the current model (Wang et al., 2014). RMSE also provided a measure of the goodness-of-fit of the model to the data used to produce it (Wang et al., 2014) and its value was 0.29 indicating that the RS model fitted well with the observed data. The statistical significance and adequacy of the model were determined using the Fisher *F*-test and Lack of Fit test (Table 4). *F*-value of 129.49 and probability value (*p* < 0.0001) indicated that the differences between different treatments were highly significant. The model is adequate since the Lack of Fit test (*p* > 0.05) was not significant (Wang et al., 2014).

**Table 4:** **Analysis of variance (*ANOVA*) for response surface model of *V. parahaemolyticus* inactivation on shelled fresh shrimp**.

**Source**	**Sum of squares**	**Degrees of freedom**	**Mean square**	***F-*value**	***P-*value**
Model	16.58	9	1.84	129.49	<0.0001
*X*_1_	0.14	1	0.14	10.06	0.0157
*X*_2_	15.43	1	15.43	484.31	<0.0001
*X*_3_	0.47	1	0.47	33.06	0.0007
$X_{1}^{2}$	0.29	1	0.29	20.23	0.0028
$X_{2}^{2}$	0.029	1	0.029	2.06	0.194
$X_{3}^{2}$	0.18	1	0.18	12.93	0.0088
*X*_1_*X*_2_	0.026	1	0.026	1.8	0.2217
_*X*_1_*X*3_	0.003025	1	0.003025	0.21	0.6587
_*X*_2_*X*3_	0.000625	1	0.000625	0.044	0.84
Residual	0.1	7	0.014		
Lack of Fit	0.082	3	0.027	6.35	0.0531
Pure error	0.017	4	0.00432		
Total	16.68	16			

**Figure 1:**
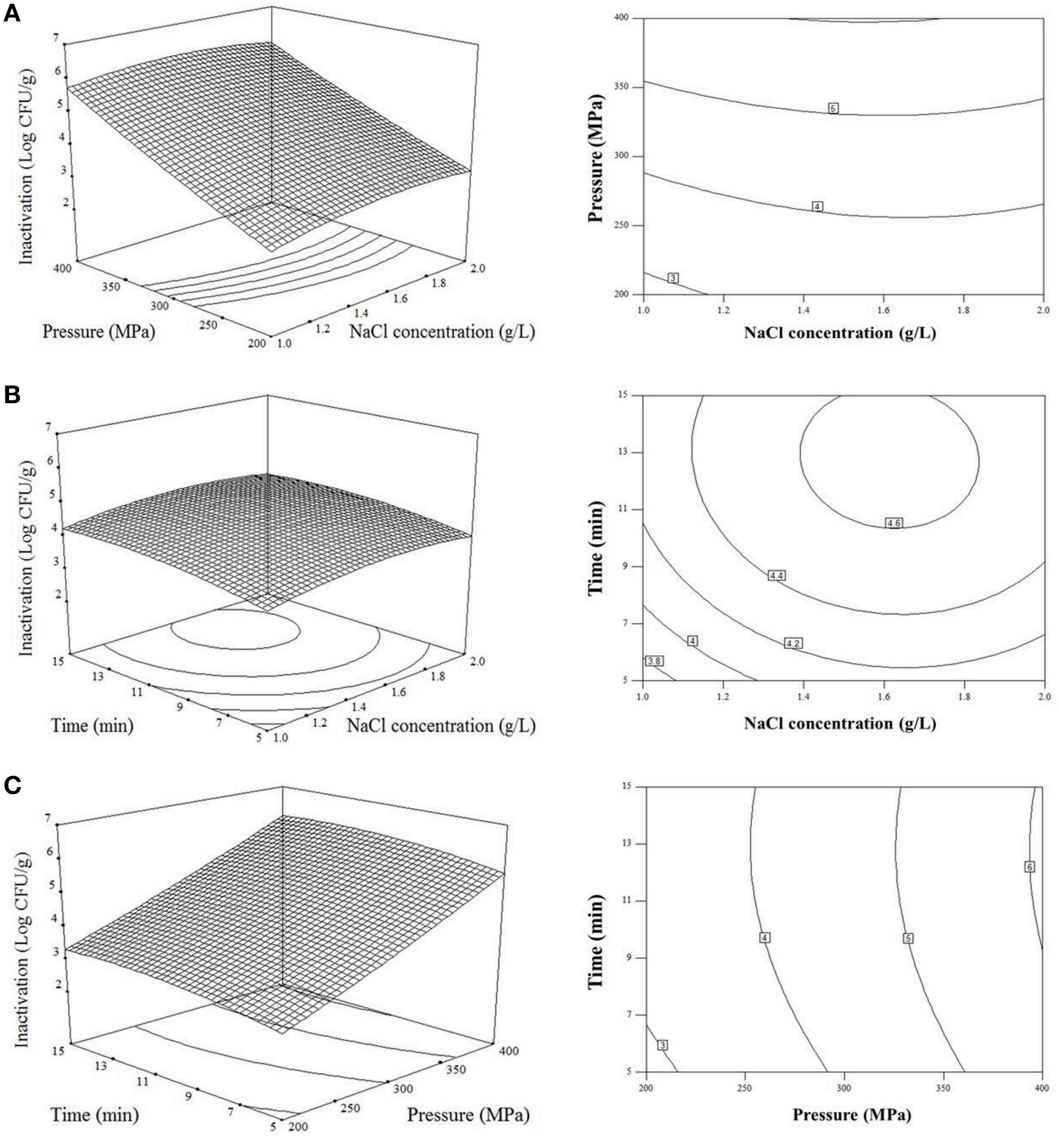
**Response surface plots describing the effect of combined high hydrostatic pressure and AEW technological variables on inactivation of *V. parahaemolyticus* on shrimps. (A)** Effects of NaCl concentration to electrolysis (*X*_1_) and pressure (*X*_2_) on reduction of *V. parahaemolyticus* with treatment time of 10 min; **(B)** Effects of NaCl concentration to electrolysis (*X*_1_) and treatment time (*X*_3_) on reduction of *V. parahaemolyticus* with pressure of 300 MPa; **(C)** Effects of pressure (*X*_2_) and treatment time (*X*_3_) on reduction of *V. parahaemolyticus* with electrolyzing NaCl concentration of 1.5 g/L.

The response surface plot in Figure 1A describes the inactivation effect of NaCl concentration and treatment pressure at the treatment time of 10 min. The population of *V. parahaemolyticus* on shelled fresh shrimp significantly reduced (*p* < 0.0001) as the concentration of NaCl and pressure increased. The change of NaCl concentration played a similar role with treatment time in *V. parahaemolyticus* inactivation, and the bacteria were less sensitive to both NaCl concentration and time than pressure (Figures 1B,C). The estimated optimal treatment conditions were 1.5 g/L NaCl concentration to electrolysis at 400 MPa for 10 min, leading to a maximum bacterial reduction of 6.08 log_10_ CFU/g.

**Figure 2:**
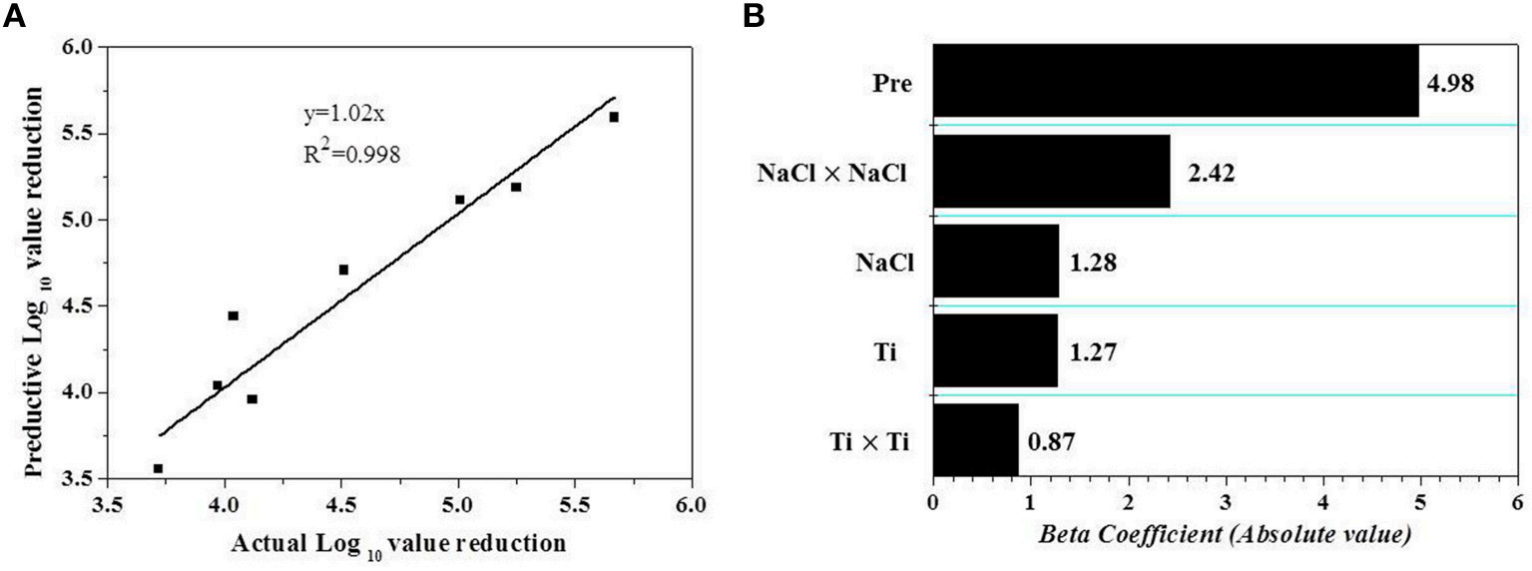
**(A)** Linear correlation between observed and predicted reductions of *V. parahaemolyticus* on shrimps under additional random 8 conditions within the range of the experimental domain. **(B)** Pareto chart of the effects of technological (independent) variables with statistical significance (*p* < 0.05) on the AEW-HHP inactivation of *V. parahaemolyticus* on shelled fresh shrimp. Beta coefficients correspond to the standardized coefficients estimated by the regression analysis. Pre and Ti are the abbreviation for pressure and time.

Validation is an important step, which can assess the capacity of developed models (Bover-Cid et al., 2012). Therefore, an external validation was carried out. The results of additional eight independent experiments (Table 3) shown in Figure 2A were used to calculate the *Af*, *Bf*, and RSME based on Equations (1–3). The *Af* value was 1.03, revealing a merely 3% difference between the observations and predictions. The calculated *Bf* was 1.01, which lies in the acceptable range of 0.9–1.05 proposed by Ross (1996). RMSE (0.19) revealed that the predicted values from RS model fitted well with the observed data.

After the establishment of the model, a Pareto chart was used to investigate the relative contribution of each variable to the *V. parahaemolyticus* inactivation (Figure 2B). The result showed that the treatment pressure was the most important factor determining the effect of AEW-HHP on inactivation of *V. parahaemolyticus* on shelled fresh shrimp within the selected variables, and the impact of the independent variable and their interactions was ranked as *X*_2_ > ${\text{X}}_{1}^{2}$ > *X*_1_ = *X*_3_ > ${\text{X}}_{3}^{2}$ according to Figure 2B.

### Effect of AEW-HHP on artificially contaminated shelled fresh shrimp

**Figure 3:**
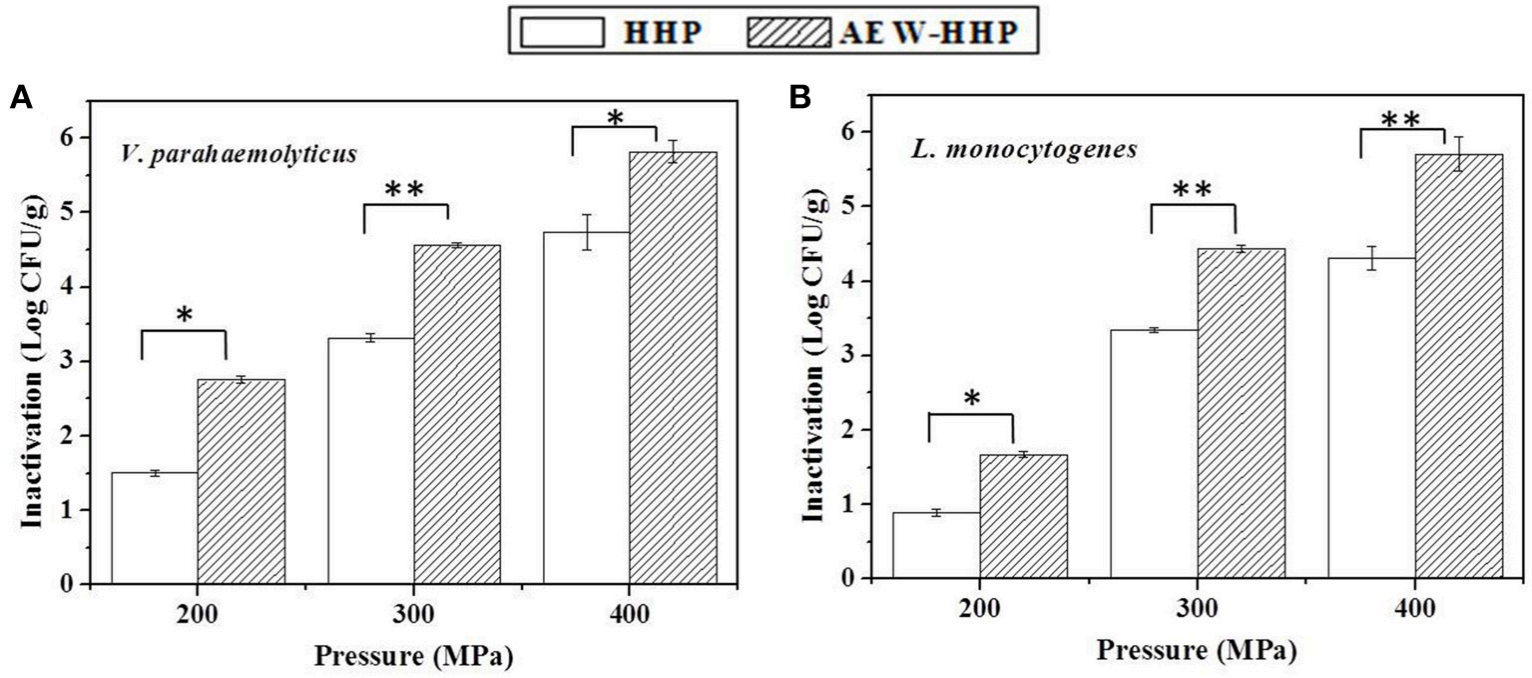
**Population reduction of *V. parahaemolyticus* (A) and *L. monocytogenes* (B) on shelled fresh shrimp after HHP and AEW-HHP for 200, 300, and 400 MPa, respectively**. Bars represent standard deviation (*n* = 3). Asterisks on the bars within the same pressure indicate significant differences (*p* < 0.05).

The initial microbial populations on shelled fresh shrimp, after inoculating and drying in a bio-safety cabinet for about 1 h, were 7.12 and 6.94 log_10_ CFU/g for *V. parahaemolyticus* and *L. monocytogenes*, respectively. Figure 3 shows the population reduction of *V. parahaemolyticus* and *L. monocytogenes* on shelled fresh shrimp after HHP and AEW-HHP at 200, 300, and 400 MPa for 10 min, respectively. There were significant differences between the microbial populations of AEW-HHP treated shrimp and those of HHP treated shrimp (*p* < 0.05). All results showed that AEW-HHP was more effective than HHP for inactivation of *V. parahaemolyticus* and *L. monocytogenes*, with a maximum reduction of about 6.08 and 5.71 log_10_ CFU/g at 400 MPa, respectively for AEW-HHP (Figure 3).

**Figure 4:**
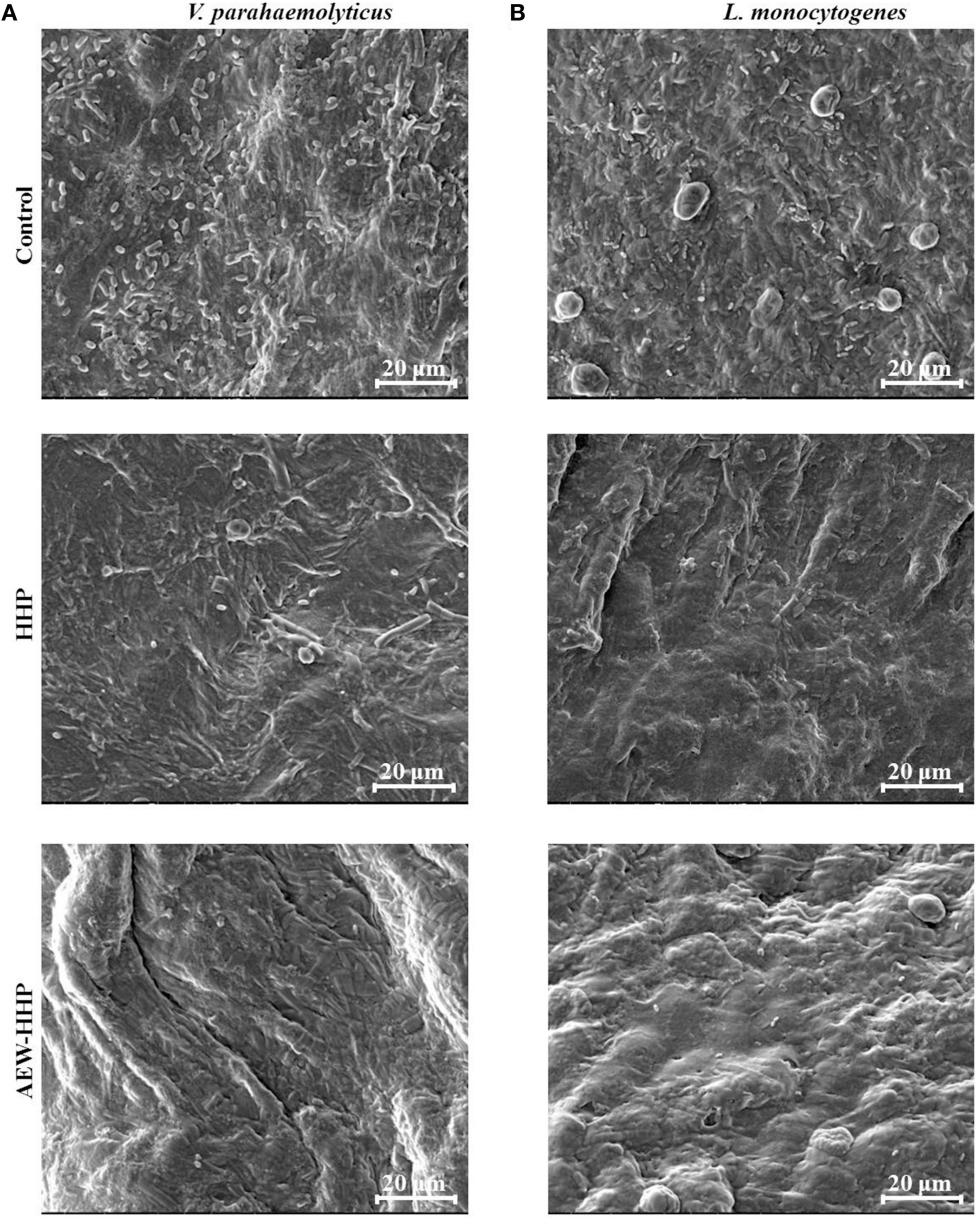
**Scanning electron microscopy (SEM) graphs of microbial populations on shelled fresh shrimp inoculated with *V. parahaemolyticus* (A) and *L. monocytogenes* (B) after different treatments**. All treatments and determinations were performed in triplicate. The treatments are as follows: untreated as control; HHP treated (NaCl concentration to electrolysis 0 g/L, treatment pressure 400 MPa, treatment time 10 min); AEW-HHP treated (NaCl concentration to electrolysis 1.5 g/L, treatment pressure 400 MPa, treatment time 10 min).

The impacts of AEW-HHP on the micromorphology of bacteria on shelled fresh shrimp were observed by SEM and the results presented in Figure 4. As shown in Figure 4, a large amount of *V. parahaemolyticus* and *L. monocytogenes* existed on the surface of the untreated shrimp samples as the control. There were still some bacterial populations on the HHP-treated shelled fresh shrimp surfaces (Figure 4), while very few microbial cells were found on the surfaces of shrimp samples after AEW-HHP treatment (Figures 4A,B). Therefore, these results showed that AEW-HHP had a remarkable efficacy on reducing the bacteria on shelled fresh shrimp.

### Effect of AEW-HHP on indigenous microflora on shelled fresh shrimp

**Figure 5:**
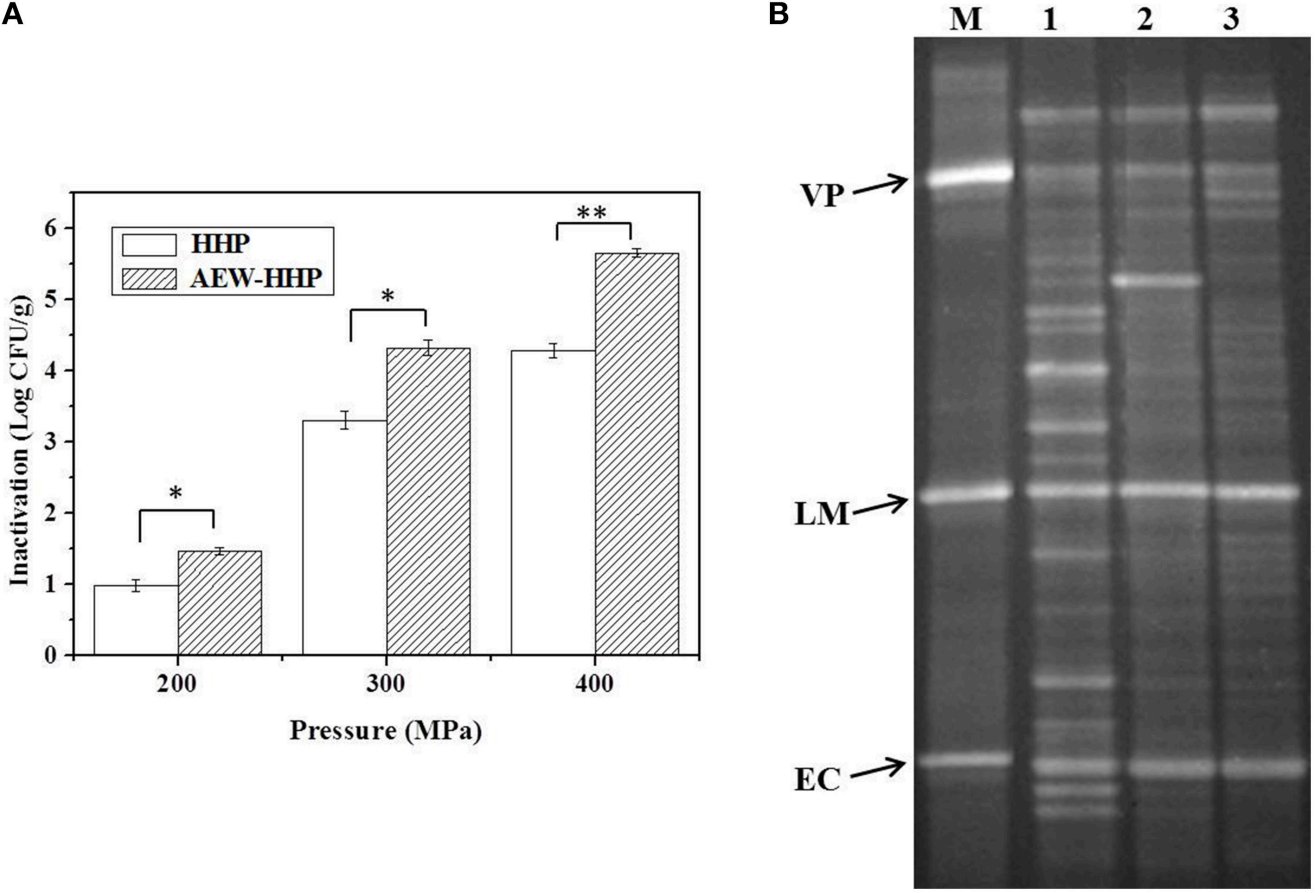
**Population reduction of natural micro flora (A) and PCR-DGGE fingerprints of bacterial flora (B) on shelled fresh shrimp after HHP and AEW-HHP treatment**. Bars represent standard deviation (*n* = 3). Asterisks on the bars within the same pressure indicate significant differences (*p* < 0.05). M, marker; 1, untreated; 2, HHP treated; 3, AEW-HHP treated; VP, *V. parahaemolyticus*; LM, *L. monocytogenes*; EC, *Escherichia coli*.

Efficacy of AEW-HHP in reducing indigenous microflora on shelled fresh shrimp is reported in Figure 5A. The initial microbial population of total aerobic bacteria in untreated fresh shelled shrimp was 6.72 log_10_ CFU/g. The micro flora decreased 0.98, 3.31, and 4.29 log_10_ CFU/g for 200, 300, and 400 MPa, respectively after HHP. In comparison with HHP, AEW-HHP treatment had obviously better disinfection efficacy. AEW-HHP at 200, 300, and 400 MPa could effectively reduce the microbial population by up to 1.47, 4.33, and 5.66 log_10_ CFU/g, respectively. There were significant differences between the microbial populations of AEW-HHP treated shrimp and those of HHP treated shrimp (*p* < 0.05).

PCR-DGGE method was carried out to investigate the effect of HHP and AEW-HHP on the diversity of microbial communities in shrimp. The results of PCR-DGGE are shown in Figure 5B. DGGE fingerprints clearly indicated that bacterial diversity in shrimp could be reduced by both HHP and AEW-HHP treatment, while AEW-HHP treatment displayed a better effectiveness than HHP. The Shannon index *H*′ of untreated shrimp samples was 2.883, and this value changed to 2.418 by HHP treatment. Surprisingly, the Shannon index *H*′ was decreased to 2.064 when AEW-HHP was applied to treat the shrimp samples. Moreover, the average similarity coefficient of DGGE fingerprints of microbial communities treated with AEW-HHP (0.567) was lower than that with HHP (0.716). Therefore, these results directly demonstrated that AEW-HHP had a stronger efficacy on reducing the diversity of microbial communities in shelled fresh shrimp.

### Effect of AEW-HHP on muscle fiber of shelled fresh shrimp

**Figure 6:**
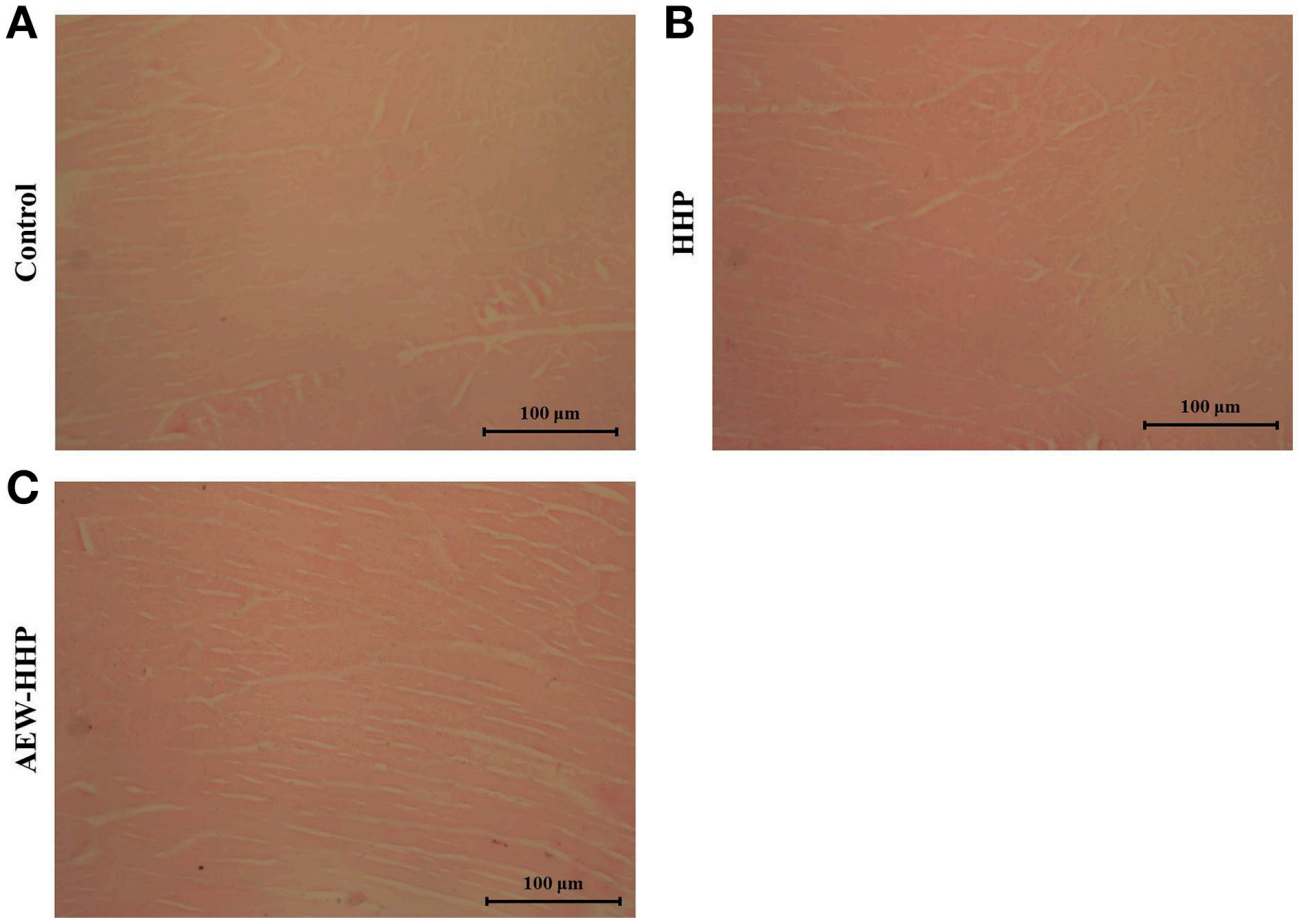
**Light microscopy observations on longitudinal sections of shrimp untreated as control (A), HHP treated (B), and AEW-HHP treated (C)**.

Figure 6 shows the light microscopic images of muscle fibers in shelled fresh shrimp treated with AEW-HHP and HHP. In the control group, the muscle fibers were tightly attached to each other and gaps between muscle fibers were much smaller with its percentage being 0.532 ± 0.043. During the subsequent treatment, the percentage of blank area was 0.478 ± 0.016 and 0.494 ± 0.022 with HHP (Figure 6B) and AEW-HHP (Figure 6C), respectively. Based on the microscopic histological analysis, AEW-HHP treatment not only could reduce natural micro flora on shelled fresh shrimp, but also had no impact on the muscle tissue of shrimp when compared with the control group (*p* > 0.05).

## Discussion

An effective sterilization and preservation technology is a prerequisite for food safety and quality in the food industry (Heinz and Buckow, 2010; Balasubramaniam et al., 2015). Developing a food preservation technology, which could markedly induce microbial inactivation and improve safety of the processed products, is necessary in the manufacturing process of food industries (Belletti et al., 2013b; Reineke et al., 2015). In the present study, we succeeded to indicate that AEW could be regarded as a new transmitting medium instead of common water for significantly improving the sterilizing effect of HHP on shelled fresh shrimp. This novel approach has great application prospect in the food industry for its excellent performance.

The optimal conditions of AEW-HHP for *V. parahaemolyticus* inactivation on shelled fresh shrimp were obtained by using a response surface methodology. The results of the model showed that 1.5 g/L was the optimal NaCl concentration to produce the AEW, and the optimal treatment pressure and treatment time were determined as 400 MPa and 10 min respectively. Under the optimized treatment conditions, this method could effectively reduce *V. parahaemolyticus* on the shelled fresh shrimp by 6.08 log_10_ CFU/g. The response surface model developed in this study gave strong evidence that AEW combined with HHP could effectively eliminate *V. parahaemolyticus* in seafood, and also this model could be applied in the future for assessing the infection risk reduction of *V. parahaemolyticus* (Espinosa et al., 2012; Praveen et al., 2013).

In order to further verify the sterilization performance of this AEW-HHP method for other bacteria, *L. monocytogenes* and indigenous microflora on shelled fresh shrimp were investigated in this study. Due to the fact that the optimal conditions for *V. parahaemolyticus* inactivation may not be appropriate for other microorganisms, we evaluated the reduction of *L. monocytogenes* and indigenous microflora on shrimp samples at 200, 300, and 400 MPa (Figures 3, 5A). Compared with common water as the pressure transmitting medium, AEW-HHP dramatically enhanced the inactivation effect for *V. parahaemolyticus* inactivation from 4.74 up to 6.08 log_10_ CFU/g. For *L. monocytogenes*, AEW-HHP could reduce 5.71 log_10_ CFU/g, while HHP could only reduce 4.31 log_10_ CFU/g. A similar result was observed for indigenous microflora on shelled fresh shrimp, with AEW-HHP showing an additional reduction of 1.37 log_10_ CFU/g. The fact that the effect of AEW-HHP was remarkably higher than that of HHP might be explained by the synergy between bactericidal techniques. As an effective sterilizing agent, AEW not only was widely used to eliminate a variety of pathogens in food products (Xie et al., 2012; Wang et al., 2014), but also has been proven to be a reliable assistant for improving the inactivation efficiency of other intervention methods (Liu R. et al., 2013; Jemni et al., 2014; Ding et al., 2015). This study firstly revealed the synergy between AEW and HHP, and further researches will be needed to investigate the mechanisms of synergy.

The microbial populations on shrimp were further observed by SEM (Figure 4). SEM has been proven as a useful tool to provide the intuitive evidences for demonstrating the survival or death of microbial populations after treatment (Hao et al., 2015; Hossain et al., 2015). In this study, the results of SEM were in agreement with those of plate counting; reduced microbial populations were found on the surfaces of inoculated *V. parahaemolyticus* and *L. monocytogenes* shrimp samples treated by AEW-HHP in comparison with HHP treatment. Although SEM is not a quantitative technique, it provided more comprehensive information and clearly displayed the survival situation of bacteria on shrimp after AEW-HHP treatment.

Besides traditional methods, molecular techniques were also employed in this study to evaluate the efficiency of AEW-HHP. DGGE fingerprints directly demonstrated that AEW-HHP had a stronger efficacy on reducing the diversity of microbial communities on shrimp than conventional HHP (Figure 5B). Previous studies have shown that microbial communities were associated with food spoilage (Sullam et al., 2012; Chaillou et al., 2015). The ability of reducing microbial diversity on shrimp indicated that this novel method could be used for prevention of seafood spoilage. Furthermore, the muscle tissues of untreated, HHP treated and AEW-HHP treated shrimp samples were compared by microscopic histological analysis. The change of muscle tissue has been demonstrated as an important index for sensory quality of food (Lin et al., 2013; Wang et al., 2015). The results showed that AEW-HHP treatment has no impact on the muscle tissue of shrimp when compared with the untreated and HHP treated groups. That indicated that this method could be regarded as an emerging preservation technology with limited effects on the sensory quality of food.

To summarize, various results of this study have demonstrated that AEW could be used as a new transmitting medium for improvement of sterilization and preservation performance of HHP processing. The AEW-HHP method could serve as a potential sterilization and preservation technology to reduce the risk of microbial infections and ensure the aquatic products safety. Hence, the AEW-HHP approach developed in this study could provide an effective tool for improving the seafood safety and protecting public health.

## Author contributions

YZ and YP designed and drafted the work, and agreed to be accountable for all aspects of the work in ensuring that questions related to the accuracy, and final approval of the version to be published. SD, ZZ, LX, and YL did the work of acquisition, analysis and interpretation of data for the work, and revising it critically for important intellectual content, and integrity of any part of the work are appropriately investigated and resolved, and final approval of the version to be published.

### Conflict of interest statement

The authors declare that the research was conducted in the absence of any commercial or financial relationships that could be construed as a potential conflict of interest.
